# The negative impact of sugar-sweetened beverages on children’s health: an update of the literature

**DOI:** 10.1186/s40608-017-0178-9

**Published:** 2018-02-20

**Authors:** Sara N. Bleich, Kelsey A. Vercammen

**Affiliations:** 1 Department of Health Policy and Management, Harvard T.H. Chan School of Public Health, Boston, MA USA; 2 Department of Epidemiology, Harvard T.H. Chan School of Public Health, Boston, USA

**Keywords:** Sugar-sweetened beverages, Children’s health

## Abstract

While sugar sweetened beverage (SSB) consumption has declined in the last 15 years, consumption of SSBs is still high among children and adolescents. This research synthesis updates a prior review on this topic and examines the evidence regarding the various health impacts of SSBs on children’s health (overweight/obesity, insulin resistance, dental caries, and caffeine-related effects). We searched PubMed, CAB Abstracts and PAIS International to identify cross-sectional, longitudinal and intervention studies examining the health impacts of SSBs in children published after January 1, 2007. We also searched reference lists of relevant articles. Overall, most studies found consistent evidence for the negative impact of SSBs on children’s health, with the strongest support for overweight/obesity risk and dental caries, and emerging evidence for insulin resistance and caffeine-related effects. The majority of evidence was cross-sectional highlighting the need for more longitudinal and intervention studies to address this research question. There is substantial evidence that SSBs increase the risk of overweight/obesity and dental caries and developing evidence for the negative impact of SSBs on insulin resistance and caffeine-related effects. The vast majority of literature supports the idea that a reduction in SSB consumption would improve children’s health.

**Electronic supplementary material:**

The online version of this article (10.1186/s40608-017-0178-9) contains supplementary material, which is available to authorized users.

## Background

Sugar sweetened beverages (SSB) – which include drinks with added sugar such as soda, fruit drinks and energy drinks – are frequently consumed by children and adolescents in the United States (U.S.) [1]. There is evidence that consumption of SSBs has recently begun to decline in the U.S., with this decrease largely driven by fewer children consuming these beverages [2, 3]. From 2003 to 2014, the percentage of children in the U.S. consuming at least one sugar-sweetened beverage on a typical day declined significantly from 80% to 61% [3]. Much of this decline was driven by a decrease in the percentage of young children ages 2 to 5 consuming SSBs, although the decline was significant for all age groups. Over the same period, consumption from caloric beverages (SSBs, milk and 100% juice) declined from 463 to 296 daily calories, and the fraction of all beverage calories from SSBs decreased from 49% to 45% [3]. Within SSBs, the number of calories from soda and fruit drinks consumed per day declined from 116 kcal to 49 kcal and 70 kcal to 31 kcal, respectively [3]. Despite these important declines, consumption of SSBs by children and adolescents in the U.S. still remains high. In 2013–2014, 46.5% of children aged 2–5, 63.5% of children aged 6–11 and 65.4% of adolescents aged 12–19 reported consuming at least one SSB on a given day [3]. Additionally, high levels of SSB consumption persist among low-income and racial and ethnic minorities.

In light of the frequent consumption of SSBs among children and adolescents in the U.S., there has been an interest in critically examining associated health consequences. As a result, there has been a substantial rise in the number of studies investigating the health effects of SSBs over the past decade. Evidence has emerged linking SSB consumption to a number of health consequences among adults including weight gain [4, 5], cardiovascular risk factors (e.g., dyslipidemia) [6], insulin resistance and type 2 diabetes [7, 8] and non-alcoholic fatty liver disease [9]. Studies among children are more limited and have generally focused on weight gain [4] and dental caries [10], as well as insulin resistance to a lesser extent [11, 12]. An emerging body of research has also examined the association between caffeinated SSBs (e.g., energy drinks or colas) and caffeine-related health consequences including reduced sleep quality and headaches [13]. Given the growing number of studies assessing SSB-related health consequences, concise summaries of the evidence base are needed in order to inform policy and advocacy efforts focused on reducing SSB consumption.

This review aims to synthesize the existing evidence regarding the impact of SSB consumption on children’s health. Unlike previous reviews which have been limited in scope (e.g., focusing on a single outcome such as weight gain) [14, 15], this review summarizes evidence from cross-sectional, longitudinal and intervention studies on a broad range of health outcomes relevant to children including: obesity, insulin resistance, dental caries, and caffeine-related effects. A previous review published in 2009 summarized many early studies on SSBs and children’s health [16]. Using a narrative review approach, we update the literature by reviewing more recent studies published up until 2017.

## Search selection

For each of the health impacts (obesity, insulin resistance, dental caries and caffeine-related effects), separate searches were conducted of PubMed, Web of Science and PAIS International. For all searches, a search hedge was created in three parts: 1) terms relevant to SSBs including “beverage” and “sodas”, 2) terms restricting to children and adolescents including “pediatric” and “teens” and 3) terms specific to the outcome being examined such as “body mass index” and “body weight” for the search on overweight and obesity risk (see Additional file 1: Appendix for full list of search terms). These search terms were chosen to retrieve the most relevant results using an iterative process in consultation with a medical librarian. For searches of PubMed, MeSH subject headings were used. In addition to database searches, reference lists of SSB reviews and articles were searched. Following the removal of duplicate studies, one author (K.V.) screened titles, abstracts and full-texts and another author (S.B.) confirmed the inclusion of these studies. Included studies had to be peer-reviewed articles examining the effects of SSBs on a specific health outcome, be limited to children and adolescents, and be published after January 1, 2007. We selected 2007 as the start date because the most recent relevant review [16] included studies published prior to this. Studies were excluded if they were not published in English, were not conducted in high-income countries (defined as membership in Organisation for Economic Co-operation and Development) or were grey literature. We limited our scope to high-income countries to promote generalizability of results.

## Effects of SSBs on health outcomes in children

### Overweight and obesity risk

A large number of studies have reported on the association between SSB consumption and overweight/obesity risk, with the majority of a cross-sectional [17–35] or longitudinal design [36–54] and only a few intervention studies (Table 1).

**Table 1:** Studies on the the overweight/obesity risk associated with SSB consumption

Author, Year	Setting	Sample Size	Sample Age	Method of Diet Assessment	SSB Unit of Analysis	Primary Outcome	Direction of Association	Findings
Cross-Sectional Studies								
Beck, 2013	Mexican American children recruited from enrollees of Kaiser Permanente Health Plan of Northern California	319	8-10 years	Youth/ Adolescent FFQ	Increment of a serving/day of soda (1 serving = 240ml)	Odds of obesity	Positive	OR = 1.29 [95%CI: 1.13, 1.47]*
Bremer, 2010A	Nationally representative sample of U.S. adolescents, NHANES, 1988-1994, 1999-2004	1988-1994: 3234 1999-2004: 6967	12-19 years	Single 24-hour dietary recall interview	Increment of a serving/day of SSB (1 serving =250g)	Change in BMI percentile for age-sex	Mixed Null for one follow-up Positive for one follow-up	1988-1994 β = 0.38 [SE: 0.45] 1999-2004 β = 0.93 [SE: 0.18]*
Bremer, 2010B	Nationally representative sample of U.S. adolescents, NHANES, 1999-2004	6967	12-19 years	Single 24-hour dietary recall interview	Increment of a serving/day of SSB (1 serving =250g)	Change in BMI percentile for age-sex	Mixed Positive in two sub-groups Null in one sub-group	Non-Hispanic White: β = 1.08 [SE: 0.21]* Mexican-American: β = 0.59 [SE: 0.29]* Non-Hispanic Black: β = 0.37 [SE: 0.26]
Clifton, 2011	Australian children as part of Australian National Children’s Nutrition and Physical Activity Survey	4400	2-16 years	Single 24-hour dietary recall interview	Consumed any amount of SSB in last 24 hours	Proportion of overweight or obese children who consumed SSBs vs. proportion of non-overweight children Proportion of obese children who consumed SSBs compared to proportion of non-overweight children	Mixed Null for one comparison Positive for one comparison	Overweight and Obese vs. Normal Weight 50% vs. 47% No measure of variation reported Obese vs. Normal Weight 59% vs. 47%* No measure of variation reported
Coppinger, 2011	British schoolchildren in south-west London, UK	248	9-13 years	Three day diary (Friday-Sunday)	mL/day of SSB	Correlation with BMI or BMI z-score	Null	No significant correlation [*r*= 0.05 for soft drinks and BMI, *r*=0.10 for fruit beverages]
Danyliw, 2012	Representative survey of Canadian children and adolescents	10,038	2-18 years	Single 24-hour dietary recall interview	Soft drink beverage cluster vs. moderate beverage pattern (mean beverage consumption in each cluster differed by gender and age group)	Odds of overweight-obesity	Mixed Positive in one sub-group Null in other sub-groups	Males, 6-11 years old OR= 2.3 [95%CI: 1.2, 4.1] * Females, 6-11 years old OR = 0.8 [95%CI: 0.4, 1.7] Males, 12-18 years old OR = 0.7 [95%CI: 0.4-1.2] Females 12-18 years old OR: 1.1 [0.6, 1.9]
Davis, 2012	Low-income Hispanic toddlers from Los Angeles WIC program, 2008 data	1483	2-4 years	Interview about early-life feeding practices and nutritional intake	No SSB vs. High SSB (≥2 SSBs/day) (1 serving = 12 ounces)	Odds of obesity	Positive	OR= 0.69 [95%CI: 0.47, 1.00]*
Davis, 2014	Low-income Hispanic toddlers from Los Angeles WIC program, 2011 data	2295	2-4 years	Interview about early-life feeding practices and nutritional intake	No SSB vs. High SSB (≥2 SSBs/day), (1 serving = 12 ounces)	Odds of obesity	Positive	AOR = 0.72 [95%CI: 0.5, 1.0]*
Denova-Gutiérrez, 2009	Adolescent children of workers at two institutes and one university in Mexico	1055	10-19 years	Semi-quantitative FFQ	Increment of a serving/day of sweetened beverage (1 serving = 240mL)	Change in BMI Odds of obesity	Positive	β =0.33 95%CI: 0.2, 0.5]* OR=1.55 [95%CI: 1.32, 1.80]*
Gibson, 2007	Children in the UK part of the UK National Dietary and Nutritional Survey of Young People	1294	7-18 years	Seven day weighed food records	Top tertile of caloric soft drink intake (>396kJ/day)) vs. bottom tertile (<163kj/day)	Odds of overweight	Weakly Positive	OR=1.39 [95%CI: 0.96, 2.0]
Grimes, 2013	Nationally representative sample of Australian children	4283	2-16 years	Two 24-hour dietary recalls	More than one serving/day vs. less than one serving/day (1 serving = 250g)	Odds of overweight-obese	Positive	OR=1.26 [95%CI: 1.03, 1.53]*
Gómez-Martinez, ^2009^	Representative sample of urban Spanish adolescents	1523	13-18 years	Single 24-hour dietary recall	Non-consumers vs. moderate consumption (<336g/day) vs. high consumption (>336g/day) of sweetened soft drinks	Mean BMI	Null	No significant differences in BMI across SSB consumption groups
Ha, 2016	Combination of 5 studies conducted on Korean children between 2002 and 2011	2599	9-14 years	Three day dietary records	More than one serving/day vs. no SSB (1 serving = 200mL)	Odds of obesity	Mixed Negative in one sub-group Null in one sub-group	Males OR: 0.52 [95%CI: 0.26, 1.05]* Females OR: 1.36 [95%CI: 0.62, 2.97]
Jiménez-Aguilar, 2009	Representative sample of Mexican adolescents who participated in Mexican National Health and Nutrition Survey	10,689	10-19 years	Semi-quantitative FFQ	Increment of a serving/day of soda (1 serving = 240ml)	Change in BMI	Mixed Positive in one sub-group Null in one sub-group	Males β =0.17 [95%CI: 0.02, 0.32]* Females β =-0.07 [95%CI: -0.23, 0.10] Note: these results are for soda. See full paper for fruit drinks, sugar beverages and SSBs.
Kosova, 2013	Nationally representative sample of U.S. children from NHANES, 1994-2004	4880	3-11 years	Single 24-hour dietary recall interview	Increment of a serving/day of SSB (1serving = 250g)	Change in BMI percentile	Mixed Null overall and in some sub-groups Positive in one sub-group	Overall β =0.71 [SE=0.38] 3-5 year olds β =-0.46 [SE=0.68] 6-8 year olds β =0.19 [SE=0.65] 9-11 year olds β =1.42 [SE=0.46]*
Linardakis, 2008	Children in public kindergartens in a single county in Greece	856	4-7 years	Three day weighed dietary records	High consumers (>250g/day) vs. non/low consumers of sugar-added beverage	Odds of obesity	Positive	OR= 2.35* No measure of variation reported
Papandreou, 2013	Greek children in Thessaloniki	607	7-15 years	Three 24-hour dietary recalls	High consumers (>360mL/day) vs. low (<180mL/day) of SSBs	Odds of obesity	Positive	OR = 2.57 [95%CI: 1.06, 3.38]*
Schröder, 2014	Representative sample of Spanish adolescents	1149	10-18 years	Single 24-hour dietary recall	Soft drink beverage cluster (mean= 553g) vs. whole milk cluster	One-unit increase in BMI z-score	Positive	Males OR = 1.29 [95%CI: 1.01, 1.65]* Note: No soft drink cluster was identified for females
Valente, 2010	Elementary school children in Portugal	1675	5-10 years	Semi-quantitative FFQ	>2 servings/day (330mL) vs. less than 1 serving/day	Odds of overweight	Null	Males OR: 0.64 [95%CI: 0.33, 1.52] Females OR: 0.63 [95%CI: 0.33, 1.22]
Longitudinal Studies								
Ambrosini, 2013	Adolescent offspring from Australian Pregnancy Cohort (Raine) Study	1433	14 years old, followed-up at 17 years old	FFQ, at baseline and follow-up	Movement into top tertile of SSB consumption (>1.3 servings/day) at follow-up vs. remaining in lower SSB tertile	Odds of overweight-obesity at follow-up	Mixed Null in one sub-group Positive in one sub-group	Males: OR: 1.2 [95%CI: 0.6, 2.7] Females OR: 4.8 [95%CI: 2.1, 11.4] *
Chaidez, 2013	Convenience sample of Latino mother and toddler pairs	67 mothers	1-2 years, followed-up for 6 months	Four 24-hour dietary recall (2 at baseline, 2 at follow-up)	High SSB consumption (higher than median) vs. low SSB consumption (lower than median)	BMI z-score, weight for height z-score, and weight for age z-score at follow-up	Mixed Positive for one measure. Null for other measures.	*Weight for height z-score* β =0.46* *BMI z-score* β =0.47 *Weight for age z-score* β =0.13 *No measure of variation reported*
DeBoer, 2013	Nationally representative sample of toddlers in the U.S.	9600	9 months, 2, 4 and 5 years (followed-up at each age)	Computer-assisted interview with questions about beverage consumption, at each follow-u	≥1 serving/day vs. <1 serving/day of SSB (1 serving = 8 ounces)	BMI z-score at follow-up (between 2 and 4 years and between 4 and 5 years)	Mixed	Measure of association not reported. Positive for change between 2 and 4 years, null for change between 4 and 5 years.
Dubois, 2007	Representative sample of children in Quebec, Canada	1944	2.5, 3.5, 4.5 years (followed-up at each age)	Single 24-hour dietary recall and FFQ at each follow-up	Regular consumers (4-6 servings/week between meals) between ages 2.5 and 4.5 years vs. non-consumers of SSBs	Odds of being overweight at follow-up	Positive	OR: 2.36 [OR: 1.10, 5.05]*
Field, 2014	Children of participants in the Nurses’ Health Study 2 in the U.S.	7559	9-16 years, followed-up for 7 years	Youth/ Adolescent FFQ, at baseline and follow-up	Increment of baseline and change in sports drink serving/day (serving =1 can)	BMI score at follow-up	Mixed	Results differed depending on type of SSB and whether predictor was baseline intake or change in intake. Results below are for sports drink intake. Females *Baseline:* β =0.29 [95%CI: 0.03, 0.54]* *Change:* β =0.05 [95%CI: =-0.19, 0.29] Males: *Baseline:* β =0.33 [95%CI: 0.09, 0.58]* *Change:* β =0.43 [95%CI: 0.19, 0.66]*
Fiorito, 2009	Non-Hispanic white girls in the U.S.	170	5 years, assessed biennially until 15 years	Three 24-hour dietary recalls at each follow-up	≥2 servings of SSB/day vs. < 1 serving of SSB/day at age 5, (1 serving = 8 ounces)	Percentage overweight in each SSB consumption group at each follow-up	Positive	5 years old ≥2: 38.5% <1: 16.1% 7 years old ≥2: 46.2% <1: 15.1 % 9 years old ≥2: 46.2% <1: 24.2% 11 years old ≥2: 53.9% <1: 21.7% 13 years old ≥2: 46.2% <1: 22.2 15 years old ≥2: 32.0 <1: 18.5 *Significant main effect
Jensen, 2013A	Danish children entering school in Copenhagen participating in intervention study	366	6, 9, 13 years (followed-up at each age)	7 day dietary record at 6 and 9 years	Increment of a serving/day of SSBs at 6 or 9 years, (1 serving = 100g)	Change in BMI from 6 to 9 years, 6 to 13 years or 9 to 13 years	Null	Intake at age 6, change from 6 to 9 years β =-0.005 [95%CI: -0.059, 0.0489] Intake at age 6, change from 6 to 13 years β =-0.059 [95%CI: -0.145, 0.027] Intake at age 9, change from 9 to 13 years β =0.008 [95%CI: -0.098, 0.113] Note: these results are for SSBs. See full paper for sweet drinks and soft drinks separately.
Jensen, 2013B	Comparison groups of two quasi-experimental intervention studies in Australia (BAEW, IYM)	1465	4-18 years, followed-up approximately 2 years later	Asked participants how much SSB consumed yesterday or last school day	Increment of a serving/day of sweet drink at baseline, (1 serving = 100mL)	BMI z-score at follow-up	Null	BAEW study: Β=0.005 [95%CI: -0.003, 0.012] IYM study: β =0.004 [95%CI: -0.002, 0.01]
Kral, 2008	Cohort of white children in U.S. born at different risks for obesity (based on maternal pre-pregnancy BMI)	49	3-6 years, followed-up at ages 3, 4, 5 and 6 years	Three day weighed food record	Change in calories from SSB from ages 3-5	Change in BMI z-score over follow-up	Null	Measure of association not reported
Laska, 2012	Adolescents enrolled in two longitudinal cohort studies in the U.S. (IDEA, ECHO)	693	6^th^ to 11^th^ grade, followed-up 2 years later	Three telephone-administered 24-hour dietary recalls	Increment of a serving/day (1 serving = not reported)	BMI at follow-up	Mixed Positive in one sub-group Null in one sub-group	Males β =0.25 [SE: 0.10]* Females β =-0.09 [SE: 0.16] Note: Above association was no longer significant when correcting for multiple testing
Laurson, 2008	Cohort of children in three rural U.S. states	268	10 years, followed-up for 18 months	Questionnaire asking about SSB consumption	SSB consumption (1 serving = not reported)	Spearman correlation with BMI at baseline or follow-up or change in BMI	Null	Males Baseline r= 0.009 Follow-up r= 0.033 Change r=0.041 Females Baseline 0.073 Follow-up 0.077 Change -0.033
Lee, 2015	Non-Hispanic Caucasian and African-American girls in the U.S.	2021	9-10 years, followed-up for 1 year	Three day food records	Increment of one teaspoon of added sugar (liquid form)	Change in BMI z-score at follow-up	Positive	β = 0.002 [95%CI: 0.001, 0.003)*
Leermakers, 2015	Dutch children in population-based prospective cohort study	2371	13 months, followed-up at ages 2, 3, 4 and 6	Semi-quantitative FFQ, validation against 24-hour recalls	High intake (15 servings/week) vs. low intake (3 servings/week) of sugar-containing beverages at 13 months, (1 serving = 150ml)	Change in BMI z-score at different follow-up ages	Mixed Null in some sub-groups Positive in other sub-groups	Males 2 year olds β =-0.01 [95%CI: -0.15, 0.12] 3 year olds β = -0.01 [95%CI: -0.15, 0.12] 4 year olds β =0.01 [95%CI: -0.12, 0.09] 6 year olds β =0.05 [95%CI: -0.08, 0.18] Females 2 year olds β =0.15 [95%CI: 0.01, 0.30]* 3 year olds β =0.14 [95%CI: 0.01, 0.27]* 4 year olds β =0.13 [95%CI: 0.01, 0.25]* 6 year olds β =0.11 [0.00, 0.23]*
Libuda, 2008	German adolescents participating in longitudinal study (DONALD)	244	9-18 years, followed-up for 5-years	Three day weighed dietary records	Baseline and change in regular soft drink consumption	BMI z-score at follow-up	Null	Males *Baseline soft drink consumption* β =0.046 *Change in baseline soft drink consumption* β =0.009 Females *Baseline soft drink consumption* β =-0.291 *Change in baseline soft drink consumption* β =0.055 *Measures of variation not reported*
Lim, 2009	Low-income African-American children	365	3-5 years, followed-up for 2 years	Block Kids FFQ	Increment of an ounce/day of SSB at baseline	Odds of incidence of overweight at 2-year follow-up	Positive	OR=1.04 [95%CI: 1.01, 1.07]*
Millar, 2014	Nationally representative cohort of Australian children	4164	4-10 years, followed-up for 6 years	Parental interview asked about SSB consumption in past 24 hours	Increment of a serving/day (serving = not reported)	Change in BMI z-score at follow-up	Positive	β =0.015 [95%CI: 0.004, 0.025]*
Pan, 2014	Children in Infant Feeding Practices Cohort Study in U.S.	1189	10-12 months, followed-up at 6 years	Survey including questions about SSB consumption	Ever consumed SSBs vs. never consumed during infancy High intake of SSBs (≥3 times/week) vs. no intake of SSBs during infancy	Odds of obesity at 6 years	Positive	*Ever Consumed vs. Never consumed:* OR: 1.71 [95%CI: 1.09, 2.68]* *High vs. No SSBs* OR: 2.00 [95%CI: 1.02, 3.90]*
Vanselow, 2009	U.S. Adolescents from various socioeconomic and ethnic background in Minneapolis/St Paul metropolitan area	2294	Adolescents, followed-up for 5 years	Youth/ Adolescent FFQ	Stratified by different number of soft drinks serving/week (0, 0.5-6, ≥6)	Change in BMI over 5-year follow-up	Null	0 servings β =1.74 [SEM= 0.18] 0.5-6 servings β =1.92 [SEM=0.10] ≥7 servings 1.80 [SEM=0.15] No significant differences across groups Note: these results are for soft drinks. See full paper for punch, low-calorie soft drinks, etc.
Weijs, 2011	Dutch children	120	4-13 months, followed-up 8 years later	Two day dietary record	Beverage sugar intake per one percent of energy intake	Odds of overweight	Positive	OR: 1.13 [95%CI: 1.03, 1.24]*
Zheng, 2014	Danish children part of European Youth Heart Study	283	9 years, followed-at ages 15 and 21	24-hour dietary recall, supplemented by qualitative food record from same day, conducted at baseline and first follow-up	≥1 serving (12 ounces) vs. none at 9 years or 15 years Increase in SSB serving from 9 to 15 years vs. no change	Change in BMI from 9 to 21 years or from 15 to 21 years	Mixed	Change in BMI from 9 to 21 years, using 9 years SSB as predictor 1.42 [SE: 0.68] Change in BMI from 15 to 21 years, using 15 years SSB as predictor 0.92 [SE: 0.54]* Change in BMI from 15 to 21 years, using change in SSB from 9 to 15 years as predictor 0.91 [SE: 0.57]
Intervention Studies								
Author, Year	Setting	Sample Size	Sample Age	Intervention	Control	Primary Outcome	Direction of Association	Findings
de Ruyter, 2012	Normal weight Dutch children	641	4-11 years	250mL sugar-free, artificially sweetened beverage	Similar sugar-containing beverage (104 calories)	Difference in change of BMI z-score from baseline at 18-month follow-up	Positive	-0.13 [95%CI: -0.21, -0.05]*
Ebbeling, 2012	Overweight and obese adolescents in U.S. who reported consuming at least 12oz of SSB/day	224	Grade 9 or 10	1-year intervention designed to decrease SSB consumption	No beverage (given supermarket gift cards as retention strategy)	Difference in change of BMI z-score from baseline to 1 year and from 1 year to 2 years (Change in experimental group minus change in control group)	Mixed	1-year follow-up -0.57 [SE: 0.28]* 2-year follow-up -0.3 [SE: 0.40]
James, 2007	Longitudinal follow-up of children involved in intervention in United Kingdom	434	7-11 years	Discouraged children from consuming SSBs and provided one hour of additional health education during each of four school terms	No beverage	Odds of overweight at 1 year and 3-years after baseline intervention (intervention ended at 1 year)	Mixed	1-year follow-up OR=0.58 [95%CI: 0.37, 0.89] * 3-year follow-up OR=0.79 [95%CI: 0.52, 1.21]

Note: *indicates statistical significance (*p*<0.05) as reported by each study

#### Cross sectional studies

Most cross-sectional studies found significant positive associations between SSB intake and obesity risk among children and adolescents [17–19, 21–25, 27, 29–32, 34, 35, 55]. For example, among 12 to 19 year olds in the 1999–2004 National Health and Nutritional Examination Survey (NHANES), each additional SSB serving (250 g) consumed per day was associated with a 0.93-percentile increase in Body Mass Index (BMI) z-score [34]. These positive findings were well-replicated across a range of OECD countries, including Canada, Spain, Greece and in Australia where those who consumed more than one SSB servings (≥250 g) per day were 26% more likely to be overweight or obese compared to those who consumed less than one serving per day [27]. They are also consistent with results focused on specific sub-groups such as among Mexican-American children aged 8–10 years where each additional SSB serving (240 mL) per week was associated with a 1.29 greater odds of obesity [17] and among toddlers living in low-income families where no SSB intake was associated with a 31% lower obesity prevalence compared to households where toddlers consumed two or more SSB servings (serving = 12 fluid ounces) per day [23].

Some of the cross-sectional studies found positive associations only within subsets of the sample [18, 19, 21, 29, 32, 35, 55], including: boys [32, 35], boys aged 6 to 11 [21], children aged 9 to 11 [29], and among Mexican-American and non-Hispanic White adolescents only [18].

A small number of cross-sectional studies reported null findings [20, 26, 33], and one study conducted in Korea among 9 to 14 year olds reported an inverse association among males [28].

#### Longitudinal studies

Like the cross-sectional data, longitudinal studies generally demonstrated that increased SSB consumption was associated with weight-related outcomes among children and adolescents [38, 39, 47–49, 51, 53, 56]. For example, among a nationally representative survey of 2 to 5 year olds in the U.S., children who consumed more than one SSB serving (serving = 8 fluid ounces) per day at 2 years old had a significantly greater increase in BMI z-score over the next 2 years compared to infrequent/non SSB drinkers [38]. Two of the positive studies examined longitudinal associations between SSB consumption and obesity risk among minority populations, with one finding that high SSB intake (defined as greater than median intake in study population) among Latino toddlers was associated with a 0.46 unit increase in weight for height z-score at 6-month follow-up [37] and the other finding that SSBs were positively associated with 2-year overweight risk among African-American preschool children [47].

Some studies found mixed results [36–38, 40, 44, 45, 52], with two reporting the positive association between SSB intake and increased weight was only significant among girls [36, 45]. The first study found high SSB intake (≥15 servings/week) at 13 months old was significantly associated with an increased BMI among girls at ages 2, 3, 4, and 6 years old [45]. Another study found that girls who moved to the top tertile of SSB consumption (>335 g/day) between 14 and 17 years of age had increased BMI and nearly a five-fold greater odds of overweight or obesity risk compared to girls who remained in the lowest tertile of SSB consumption [36]. One study found a positive association when using SSB consumption at 15 years to predict change in BMI from ages 15–21 and found null results when using SSB consumption at 9 years as a predictor [52].

Some of the longitudinal studies found no association between SSBs and BMI or BMI z-scores [41–44, 46, 50, 54, 57].

#### Intervention studies

A small number of intervention studies have examined SSB consumption and overweight and obesity risk among children [58–60]. Three recent randomized controlled trials found a reduction in BMI or obesity risk in the intervention group compared to the control. De Ruyter and colleagues conducted a double-blinded placebo-controlled trial wherein 641 normal weight Dutch children were randomized to receive either a 250 mL of an SSB or a sugar-free beverage each day for 18 months [58]. At the end of the trial, the difference in BMI z-score was significantly different between the two groups, with the SSB group increasing on average by 0.15 units (compared to 0.02 units in the sugar-free group). The second study randomized 224 overweight and obese American adolescents who regularly consumed SSBs to either participate in a program to reduce SSB consumption or receive no intervention [59]. At the end of the 1-year intervention, those in the intervention group had beneficial changes in BMI and weight compared to those who did not receive the intervention, but these differences were no longer significant when participants were followed-up for an additional year after the end of the intervention. However, in a pre-planned subgroup analysis of Hispanic participants, there were significant differences in BMI between groups at both follow-up periods. The third study was a cluster randomized trial in which schools in the United Kingdom were randomized to either an intervention discouraging consumption of SSBs or no intervention for one year [61]. A significant difference in BMI z-score and overweight/obesity risk between groups was observed at the end of the first year, supporting a positive association between SSBs and obesity risk [61]. Two years after the intervention had been discontinued, the researchers completed a follow-up assessment and reported the differences between the groups were no longer significant [60].

### Insulin resistance

A modest number of studies reported a positive association between SSB consumption and insulin resistance risk among children and adolescents, with the majority conducted cross-sectionally [62–65], one conducted longitudinally [66] and no intervention studies conducted (Table 2).

**Table 2:** Studies on the insulin resistance risk associated with SSB consumption

Author, Year	Setting	Sample Size	Sample Age	Method of Diet Assessment	SSB Unit of Analysis	Primary Outcome	Direction of Association	Findings
Cross-Sectional Studies								
Bremer, 2009	Nationally representative sample of U.S. adolescents, NHANES, 1994-2004	6967	12-19 years	Single 24-hour dietary recall interview	Increment of a serving/day (serving = 250g)	Change in HOMA-IR	Positive	β = 0.05 [SE= 0.02]*
Bremer, 2010	Nationally representative sample of U.S. adolescents, NHANES, 1999-2004	6967	12-19 years	Single 24-hour dietary recall interview	Increment of a serving/day (serving = 250g)	Change in HOMA-IR	Mixed	Non-Hispanic White: β= 0.06 [SE=0.02]* Non-Hispanic Black: β=0.12 [SE=0.05]* Mexican Americans: β=0.04 [SE=0.04]
Kondaki, 2012	Adolescents in large multicenter European study	546	12-17 years	Mini FFQ from Health Behavior in School-Aged Children study	≥1 time/day vs. <1 time/week 5-6 times/week vs. <1 time/week 2-4 times/week vs. <1 time/week, (serving = not reported)	Change in HOMA-IR	Positive	≥1 time/day vs. ≤ 1 time/week β = 0.19 [95%CI: 0.003, 0.38]* 5-6 times/week vs. ≤1 time/week β = 0.28 [95%CI: 0.07, 0.49]* 2-4 times/week vs. ≤ 1 time/week β =0.080 [95%CI: -0.084, 0.245]
Santiago-Torres, 2016	Hispanic children attending inner-city school in Milwaukee	187	10-14 years	Block for Kid’s FFQ with Hispanic foods	SSB consumption, (serving = not reported)	Change in HOMA-IR	Positive	β =0.104* *No measure of variation reported*
Wang, 2012	Caucasian children recruited from primary schools in Canada	632	8-10 years	Three 24-hour dietary recalls	Increment of a serving/day (serving = 100ml)	Change in HOMA-IR	Mixed Null overall Positive in one sub-group Null in one sub-group	Among all children: β =0.024 > 85^th^ BMI percentile β = 0.097* <85^th^ BMI percentile β =-0.027 *No measure of variation reported*
Longitudinal Studies								
Wang, 2014	Caucasian Canadian children with at least one obese parent	564	8-10 years	Three 24-hour dietary recalls	Increment of 10g/day of added sugar from liquid sources	HOMA-IR	Positive	Among all children: 0.091 [95%CI: 0.034, 0.149] * Overweight/ obese: 0.121 [95%CI: 0.013, 0.247] * Normal weight: 0.046 [95%CI: -0.003, 0.096]

Note: *indicates statistical significance (*p*<0.05) as reported by each study

#### Cross sectional studies

A number of cross-sectional studies found a positive association in the whole or a subset of their study population [62–65]. For example, among 12–19 year olds in NHANES, each additional SSB serving (250 g) consumed per day was associated with a 5% increase in HOMA-IR (a marker of insulin resistance which is calculated using fasting glucose and insulin levels) [55]. One study reported associations by race, with positive associations found among White and African Americans, but null associations among Mexican Americans [18]. Another study reported a stronger association between SSB consumption and higher HOMA-IR among overweight/obese participants compared to normal weight participants [64].

#### Longitudinal studies

Only one longitudinal study was conducted to examine this association, reporting that an additional 10 g/day of added sugar from liquid sources was associated with a 0.04 mmol/L higher fasting glucose, 2.3 pmol/L higher fasting insulin and a 0.01 unit increase in HOMA-IR over two year follow-up [66].

### Dental caries

A growing number of studies have examined the relationship between SSB consumption and dental caries (cavities or tooth decay) among children and adolescents, with almost all evidence pointing towards a strong positive association (Table 3). While the majority of studies examining SSB intake and dental caries are cross-sectional [67–82], there have been several longitudinal studies [83–88] and one intervention study [89].

**Table 3:** Studies on the dental caries risk associated with SSB consumption

Author, Year	Setting	Sample Size	Sample Age	Method of Diet Assessment	SSB Unit of Analysis	Primary Outcome	Direction of Association	Findings
Cross-Sectional Studies								
Armfield, 2013	Australian children enrolled in school dental services	16,508	5-16 years	Questionnaire given to parents asked about SSB consumption	≥3/day, 1-2/day vs. 0/day, (1 serving = “1 medium glass”)	Decayed, missing and filled deciduous teeth (for ages 5-10) Decayed, missing and filled permanent teeth (for ages 11-16)	Positive	5-10 years old *≥3 vs. 0 servings/day* β = 0.46 [95%CI: 0.29, 0.64]* 1-2 vs. 0 servings/day β = 0.34 [95%CI: 0.23, 0.45]* 11-16 years old *≥3 vs. 0 servings/day* β = 0.27 [95%CI: 0.13, 0.41]* 1-2 vs. 0 servings/day β = 0.16 [95%CI: 0.06, 0.26]*
Chi, 2015	Convenience sample of Alaska Native Yup’ik children	51	6-17 years	Verbally administered survey, including questions on beverage consumption adapted from Beverage and Snack Questionnaire	40 grams/day of added sugar (i.e. amount of sugar in 12-ounce soda) measured using hair biomarker and self-report. Note: Biomarker would include all sources of added sugar, not just liquid.	Proportion of carious tooth surfaces	Mixed	Biomarker: 6.4% [95%CI: 1.2, 11.6%]* Self-Report: Null. No measure of association reported.
Derlerck, 2008	Preschool children in four distinct geographical areas of Belgium	2533	3 and 5 year olds	Questionnaire given to parents with structured open-ended questions about dietary habits	Daily or more consumption of SSBs at night vs. none Daily consumption of SSBs between meals vs. none	Odds of caries experience (using criteria from British Association for the Study of Community Dentistry)	Positive	SSB consumption at night 3 year-olds OR= 7.96 [95%CI: 1.57, 40.51] * 5 year-olds OR = 1.64 [95%CI: 0.18, 14.63] SSB consumption between meals 3 year-olds OR=1.47 [95%CI: 0.36, 6.04] 5-year olds OR= 2.60 [95%CI: 1.16, 5.84] *
Evans, 2013	Low-income children recruited from pediatric dental clinics in D.C. and Ohio	883	2-6 years	Parent-completed 24-hour recall and interviewer-administered FFQ	Using 24-hour recall 1.7 to 14 servings SSB/day vs. 0 servings/day Using FFQ 0.63 to 7 servings SSB/day vs. <0.16 servings/day (1 serving = 8 ounces)	Odds of severe early childhood caries	Positive	Using 24-hour recall OR = 2.02 [95%CI: 1.33, 3.06]* Using FFQ OR = 4.63 [95%CI: 2.86, 7.49]*
Guido, 2011	Children from small rural villages in Mexico	162	2-13 years	Questionnaire with questions about beverage consumption specific to ones sold in local stores	Drinking soda at least onece/day	Decayed, missing and filled deciduous teeth Decayed, missing and filled permanent teeth	Positive	No measures of association reported p=0.71 p=0.04*
Hoffmeister, 2015	Random sample of children in southern Chile from a daycare center register	2987	2 and 4 years	Survey filled out by parents with questions about sugary drink frequency	>3 servings of sugary drinks/week at bedtime vs. ≤ 3 servings of sugar drinks/week at bedtime (1 serving = not reported)	Prevalence ratio of decayed, missing and filled deciduous teeth	Positive	2 year olds PR = 1.43 [95%CI: 0.97, 2.10] * 4 year olds PR = 1.30 [95%CI: 1.06, 1.59] *
Jerkovic, 2009	Children recruited from primary schools in northern region of the Netherlands, including low and high SES schools	301	6 and 10 years	Questionnaire filled out by parents including information on nutritional care	≥5 glasses of fruit juice/soft drinks vs. ≤4 glasses of fruit juice/soft drinks	Prevalence of caries	Positive	Measures of association not reported. p<0.001 *
Jurzak, 2015	Pediatric patients from university dental clinic in Poland	686	1-6 years	Questionnaire including questions about SSB consumption	Frequent consumption of fruit juices and carbonated drinks vs. Infrequent consumption (1 serving = not reported)	Odds of decayed, missing and filled teeth	Mixed, depending on age	1-2 years old 2.60 [95%CI: 0.77, 8.74] 3-4 years old 2.23 [95%CI: 1.25, 3.96] * 5 years old OR=2.134 [95%CI: 0.84, 5.44] 6 years old OR= 2.25 [95%CI: 1.03, 4.92]*
Kolker, 2007	African American children with household incomes below 250% of the 2000 federal poverty level	436	3-5 years	Block Kids FFQ	Consumption of soda (1 serving = not reported)	Odds of higher score of decayed, missing and filled deciduous teeth	Null	OR = 1.00 [95%CI: 1.0, 1.1] Note: this result is for soda. See full paper for powdered drinks, sports drinks, fruit drinks, etc.
Lee, 2010	Convenience sample of healthy primary school children in Australia	266	4-12 years	Prat Questionnaire asked about consumption of sweet drinks	Sweet drinks consumed in the evening/night vs. no sweet drinks consumed	Caries experience in past 12 months	Positive	18% vs. 29% p=0.004* Measure of association not reported.
Majorana, 2014	Italian toddlers born to mothers attending two obstetric wards	2395	24-30 months	Self-administered questionnaire for mothers with questions about SSB consumption	≥2 servings day vs. ≤1 servings of SSBs, (1 serving = 250mL)	Odds of higher International Caries Detection and Assessment System score	Positive	OR = 1.18 [95%CI: 0.99-1.40]*
Mello, 2008	Sample of schoolchildren in Portugal	700	13 years	Semi-quantitative FFQ	≥2 servings/week vs. ≤2 servings/week of soft drinks derived from cola, other soft drinks and any soft drinks (1 serving = not reported)	Odds of ≥4 decayed, missing and filled teeth	Positive	Soft drinks from cola OR = 2.23 [95%CI: 1.50, 3.31]* Other soft drinks OR = 1.54 [95%CI: 1.05, 2.26]* Any soft drinks OR = 1.88 [95%CI: 1.07, 3.29]*
Nakayama, 2015	Japanese infants	1675	18-23 months	Questionnaire for parents or guardian with questions about SSB consumption	Drinking soda ≥4 times/week vs. <4 times/week, (1 serving = not reported)	Odds of early childhood caries	Positive	OR = 3.70 [95%CI: 1.07, 12.81] *
Pacey, 2010	Inuit preschool-aged children in Nunavut, Canada	388	3-5 years	Past-month qualitative FFQ, 24-hour dietary recall (with repeat 24-hour recalls on 20% of sub-sample)	Mean SSB consumption compared between groups of Reported Caries Experience	Reported Caries Experience (RCE)	Positive	Mean SSB consumption /day among those with RCE 0.8 [SE=0.1] Mean SSB consumption /day among those without RCE 0.5 [SE=0.1] *Significant difference between groups.
Skinner, 2015	Random sample of adolescents in Australia	1187	14 to 15 years	Questionnaire including questions about SSB consumption	0 cup of soft drinks or cordial vs. 1-2 cups per day vs. 3+ cups per day	Mean decayed, missing and filled permanent teeth	Positive	0 cups per day Male: 1.14 Female: 0.81 1-2 cups per day Male: 1.12 Female: 1.47 3+ cups per day Male: 1.69 Female: 1.39 *Significant difference between groups. Measure of variation not reported Note: this result is for soft drinks or cordial. See full paper for sweetened fruit juice, diet soft drinks and sports drinks.
Wilder, 2016	School-based sample of third grade students in Georgia, U.S.	2944	8 and 9 years	Supplemental survey including questions about SSB consumption	Increment of a serving/day of SSB, (1 serving = not reported)	Prevalence ratio of caries experience	Positive	PR: 1.22 [95%CI: 1.13, 1.32]*
Longitudinal Studies								
Lim, 2008	Low-income African American children in Detroit	369	3-5 years, followed-up 2 years later	Block Kids FFQ	Change from low SSB consumption cluster to high SSB consumption cluster vs. low consumers at both time periods	Incident decayed, missing and filled deciduous teeth and incident filled surfaces at follow-up	Positive	*New d*_*2*_ *mfs:* IRR=1.75 [95%CI: 1.16, 2.64]* *New filled surface:* IRR=2.67 [95%CI: 1.36, 5.23]*
Park, 2015	U.S. children in Infant Feeding Practices Study II and Follow-up Study	1274	10-12 months, followed-up at 6 years of age	10 postpartum surveys through infancy, which asked about intake of SSBs during past 7 days	Any SSBs vs. no SSBs during infancy SSB introduction at or after 6 months, SSB introduction before 6 months vs. Never consumed SSBs during infancy SSB consumption < 1 time/week, 1-3 times/week, ≥3 times/week vs. No SSBs	Dental caries in child’s lifetime at follow-up	Mixed	Any vs. No intake during infancy OR = 1.14 [95%CI: 0.82, 1.57] SSB intro at or after 6 months vs. no SSB OR = 1.07 [95%CI: 0.76, 1.52] SSB intro before 6 months vs. no SSB OR = 1.29 [95%CI: 0.77, 2.17] Consumed <1 time/week vs. No SSBs during infancy OR = 1.15 [95%CI: 0.61, 2.18] Consumed 1-3 times/week vs. No SSBs during infancy OR = 0.85 [95%CI: 0.48, 1.49] Consumed ≥3 times/week vs. No SSBs during infancy OR = 1.83 [95%CI: 1.14, 2.92]*
Warren, 2009	Children in rural community in Iowa enrolled in WIC program	212	6-24 months, followed-up 9 and 18 months later	Questionnaire asking about SSB consumption at each follow-up	SSB consumption vs. no SSB consumption at baseline	Odds of caries at 18-month follow-up	Positive	OR = 3.0 [95%CI: 1.1, 8.6]*
Warren, 2016	American Indian infants from Northern Plains Tribal community	232	Infants followed-up at 4, 8, 12, 16, 22, 28 and 36 months	Validated beverage frequency questionnaire for parents adapted from Iowa Fluoride study, a 24-h dietary recall tool and food habit questionnaire	Added-sugar beverage intake as proportion of total	Odds of caries experience at follow-up	Positive	OR = 1.02 [95%CI: 1.00, 1.04]*
Watanabe, 2014	Japanese infants recruited from Kobe City Public Health Center	31,202	1.5 years, followed-up 21 months later (at ~3 years old)	Questionnaire for parents asking about SSB consumption and frequency	Daily SSB consumption vs. no SSB consumption, at baseline	Odds of dental caries at 3-years	Positive	OR = 1.56 [95%CI: 1.46, 1.65]*
Wigen, 2015	Children in the Norwegian Mother and Child Cohort Study	1095	1.5 years, followed-up at 5 years old	Questionnaire for parents asking about SSB consumption	SSBs offered at least once a week vs. less than once a week, at 1.5 years	Odds of decayed, missing and filled deciduous teeth	Positive	OR = 1.8 [95%CI: 1.1, 2.9]*
Intervention Studies								
Author, Year	Setting	Sample Size	Sample Age	Intervention	Control	Primary Outcome	Direction of Association	Findings
Maupomé, 2010	American Indian toddlers in U.S.	Four geographically separate tribal groups (3 intervention groups, 1 control group); Group A = 63 enrolled, 53 completed. Group B = 62 enrolled, 56 completed; Group C = 80 enrolled, 69 completed. Group D = NR.	18-30 months,	3-pronged approach: 1) increase breastfeeding, 2) limit SSB consumption, 3) promote consumption of water for thirst Each intervention group measured at pre and post; also compared to control group to account for secular trends	No intervention received.	Post-pre difference in fraction of affected mouths by incident caries (d1t and d2t)	Positive	*d1t* Group A: -0.574 [SDE: 0.159]* Group B: -0.300 [SDE: 0.140]* Group C: -0.631 [0.157]* *d2t* Group A**:** -0.449 [SDE: 0.180]* Group B: -0.430 [SDE: 0.153]* Group C: -0.342 [SDE: 0.181]

Note: * indicates statistical significance (*p*<0.05) as reported by each study

#### Cross sectional studies

The vast majority of cross-sectional studies found evidence for a positive association between SSB consumption and dental caries [67, 69–82]. For example, one study reported that the prevalence of caries was 22% higher for each additional SSB serving consumed by children per day [81]. Several studies replicated this positive association among low-income children [70, 73, 75], with one study reporting that high SSB consumption (≥5 oz/day) was associated with a 4.6 greater odds of dental caries compared to those with lower SSB consumption [70]. Some studies examined how specific timing of SSB consumption affects dental caries, with one study [72] finding an association with dental caries and SSBs consumed at bedtime and another [69] finding an association with dental caries and SSBs consumed at nighttime among 3 year-olds and for SSBs consumed between meals among 5-year olds.

One cross-sectional study reported null results, finding no association between self-reported SSB consumption and dental caries among Alaska Natives – a result which may have been related to the small sample size (*N* = 51) [68].

#### Longitudinal studies

All longitudinal studies included in this review found a positive or mixed association between SSB consumption and dental caries in at least part of the study population [83–88]. One study reported that a high consumption of SSBs (≥3 servings per week) among infants 10 to 12 months old was associated with a 1.83 greater odds of dental caries at age 6, compared with infants who did not consume SSBs during infancy [84]. Some studies reported these positive findings among specific subgroups including: low-income [86], African American [83] and American Indian children [85]. For example, Lim et al. conducted a cluster analysis and reported that African American children who changed from being low consumers of SSBs at baseline (mean consumption = 567.4 mL/day) to high consumers of SSBs at 2-year follow-up (mean consumption = 1032.4 mL/day) had a 1.75 times higher mean number of new dental caries compared with high consumers of milk-juice at both baseline and 2-year follow-up [83].

#### Intervention studies

Only one intervention study has been conducted to assess SSB consumption and dental caries [89]. Maupomé et al. conducted community-wide interventions to reduce SSB consumption, improve breastfeeding practices, and promote consumption of water for thirst among American Indian toddlers. While the intervention communities demonstrated improvements in the number of dental caries, it is not possible to attribute this specifically to reduction in SSB consumption as the intervention was a multi-pronged approach.

### Caffeine-related effects

A growing number of studies reported on the caffeine-related effects associated with SSB consumption with studies almost exclusively cross-sectional (Table 4).

**Table 4:** Studies on caffeine-related effects associated with SSB consumption

Author, Year	Setting	Sample Size	Sample Age	Method of Diet Assessment	SSB Unit of Analysis	Primary Outcome	Direction of Association	Findings
Cross-Sectional Studies								
Azagba, 2014	Adolescents attending public schools in Atlantic Canada	8210	Grades 7, 9, 10 and 12	Self-reported survey with question asking about consumption of caffeinated energy drinks in past year	Energy drink more than once a month vs. one to two times	Odds of depression, sensation seeking, substance use	Positive	*Sensation Seeking* OR = 1.17 [95%CI: 1.11, 1.22]* *Depressive symptoms, very elevated* OR = 1.95 [95%CI: 1.36, 2.79]* *Depressive symptoms, somewhat elevated* OR = 1.08 [95%CI: 0.80, 1.47] *Cigarette use* OR = 2.58 [95%CI: 1.71, 3.89]* *Marijuana use* OR = 1.87 [95%CI: 1.37, 2.56]* *Alcohol use* OR = 2.48 [95%CI: 1.83, 3.36]* *Other drug use* OR = 1.80 [95%CI: 1.26, 2.57]*
Bashir, 2016	Convenience sample of patients in waiting areas of emergency department in U.S.	612	12-18 years	Questionnaire asking about frequency of energy drink consumption	Frequent (at least once a month) vs. Infrequent (less than once a month) consumers of energy drinks	Proportion of frequent vs. infrequent consumers experience of headache, anger and increased urination	Positive	*Headache* 76% [95%CI: 69-81] vs. 60% [95%CI: 55-64]* *Anger* 47% [95%CI: 40-54] vs. 32% [95%CI: 27-36]* *Increased urination* 24 [95%CI: 18-30] vs. 13 [95%CI: 10-16]* Study provides a number of outcomes. See paper for full results.
Koivusilta, 2016	Classroom survey of 7^th^ grade students in Finland	9446	13 years	Self-reported online survey asking about frequency of energy drink consumption	Several times a day vs. not at all	Odds of headache, sleeping problems, irritation, tiredness/fatigue, late bedtime	Positive	*Headache* OR = 4.6 [95%CI: 2.8, 7.7] *Sleeping problems* OR = 3.6 [95%CI: 2.2, 5.8] *Irritation* OR= 4.1 [95%CI: 2.7, 6.1] *Tiredness/ fatigue* OR=3.7 [95%CI: 2.4, 5.7] *Late bedtime* OR = 7.9 [95%CI: 5.7, 10.9]
Kristjansson, 2013	School survey of children in Iceland	11,267	10-12 years	Questions on population-based survey asking about frequency of energy drink and cola consumption	≥1 cola/day vs. none ≥1 energy drink/ day vs. none	Odds of headaches, stomachaches, sleeping problems, low appetite	Positive	Colas *Headaches* Females: OR = 1.13 [95%CI: 0.87, 1.47] Males: OR = 1.29 [95%CI: 1.03, 1.62]* *Stomachaches* Females: OR = 1.40 [95%CI: 1.08, 1.80]* Males: OR = 1.31 [95%CI: 1.03, 1.67]* *Sleeping problems* Females: OR = 1.55 [95%CI: 1.21, 1.98]* Males: OR = 1.34 [95%CI: 1.09, 1.66]* *Low appetite* Females OR = 1.37 [95%CI: 1.03, 1.83]* Males OR = 1.44 [95%CI: 1.12, 1.86]* Energy Drinks *Headaches* Females: OR = 1.68 [95%CI: 1.17, 2.41]* Males: OR = 1.87 [95%CI: 1.43, 2.46]* *Stomachaches* Females: OR = 1.76 [95%CI: 1.21, 2.54]* Males: OR = 2.45 [95%CI: 1.86, 3.23]* *Sleeping problems* Females: OR = 1.56 [95%CI: 1.07, 2.25]* Males: OR = 1.63 [95%CI: 1.25, 2.12]* *Low appetite* Females OR = 2.31 [95%CI: 1.58, 3.39]* Males OR = 1.30 [95%CI: 0.95, 1.78]
Park, 2016	Nationally representative cohort of Korean adolescents	68,043	12-18 years	Web-based survey with questions on energy drink consumption	Highly frequent energy drink consumer (≥5 times/week) vs. infrequent energy drink consumer (<1 time/week) Moderate frequent energy drink consumer (1-4 times/week) vs. infrequent energy drink consumer	Odds of sleep dissatisfaction, perceived stress, persistent depressive mood, suicidal ideation, suicide plan, suicide attempt	Positive	Highly frequent energy drink consumer vs. infrequent energy drink consumer *Sleep dissatisfaction* OR = 1.64 [95%CI 1.61, 1.67]* *Perceived stress* OR = 2.23 [95%CI: 2.19, 2.27]* *Depressive mood* 2.59 [95%CI: 2.54, 2.65]* *Suicidal ideation* 3.14 [95%CI: 3.07, 3.21]* *Suicidal plan* 4.65 [95%CI: 4.53, 4.78]* *Suicide attempt* 6.79 [95%CI: 6.59, 7.00]* Moderate frequent energy drink consumer vs. infrequent energy drink consumer *Sleep dissatisfaction* OR = 1.25 [95%CI: 1.25, 1.26]* *Perceived stress* OR = 1.38 [95%CI: 1.37, 1.39]* *Depressive mood* OR=1.51 [95%CI: 1.49, 1.52]* *Suicidal ideation* OR=1.43 [95%CI: 1.42, 1.45]* *Suicidal plan* OR=1.78 [95%CI: 1.75, 1.81]* *Suicide attempt* OR=1.91 [95%CI: 1.87, 1.95]*
Richards, 2015	Adolescents from three secondary schools in the South West of England	2307	11-17 years	DABS survey (assesses intake of common dietary variables), including questions on energy drink and cola consumption	High consumption (≥1 can of energy drink or cola) vs. no consumption Low consumption (<1 can of energy drink or cola) vs. no consumption	Odds of stress, anxiety and depression	Mixed	High consumption vs. no consumption Energy Drinks *Stress* OR = 1.10 [95%CI: 0.80, 1.50] *Anxiety* OR = 1.05 [95%CI: 0.77, 1.43] *Depression* OR = 1.11 [95%CI: 0.81, 1.52] Cola *Stress* OR = 0.68 [95%CI: 0.52, 0.90]* *Anxiety* 0.83 [95%CI: 0.64, 1.09] *Depression* 1.23 [95%CI: 0.93, 1.62] Low consumption vs. no consumption Energy Drinks *Stress* 1.38 [95%CI: 1.05, 1.80]* *Anxiety* 1.26 [95%CI: 0.97, 1.64] *Depression* 0.99 [95%CI: 0.76, 1.31] Cola *Stress* 0.72 [95%CI: 0.56, 0.94]* *Anxiety* 0.86 [95%CI: 0.67, 1.10] *Depression* 1.18 [95%CI: 0.91, 1.54]
Longitudinal Studies								
Marmorstein, 2016	Cohort of middle-school students in the U.S.	144	10-14 years, followed-up 16 months later	Self-reported questionnaire with questions on energy drink consumption	Energy drink consumption at baseline	Change in ADHD inattention, ADHD hyperactive, conduct disorder, depression, panic, anxiety at follow-up (controlling for coffee)	Mixed	*ADHD inattention* β = 0.20* *ADHD hyperactive* β = 0.20* *Conduct disorder* β = 0.18 *Depression* β = 0.08 *Panic* β = 0.17 *Generalized anxiety* β = 0.09 *Social Anxiety* β = -0.02

Note: * indicates statistical significance (*p*<0.05) as reported by each study

#### Cross sectional studies

A number of cross-sectional studies examined the effects of energy drink consumption among children and adolescents [90–97], with each study often reporting on multiple outcomes. Some studies found evidence for an association between energy drink consumption and sleep-related issues such as sleep dissatisfaction, tiredness/fatigue and late bedtime [92, 93, 95], and others reported an association between energy drink intake and increased headaches [91–93]. One study reported an association between energy drink consumption and risk-taking behaviors such as cigarette, marijuana and drug use [90], and two studies found an association between energy drink consumption and stress, depressive symptoms, and suicidal ideation, plan or attempt [90, 95]. Other outcomes examined in these cross-sectional studies reported include irritation [92], stomach ache and low appetite [93].

Some of the cross-sectional studies examined caffeine-related effects of cola drinks [93, 96, 97]. One found that both low and high consumption of cola were associated with lower stress and found null associations with anxiety and depression [96]. Another examined both cola and energy drinks and found that higher consumption of both beverages was associated with headaches, stomach-aches, sleeping problems and low appetite [93]. More specifically, among males, drinking more than one cola per day was associated with a 1.34 greater odds of sleeping problems and among females drinking more than one cola per day was associated with a 1.55 greater odds of sleeping problems.

#### Longitudinal studies

One longitudinal study was conducted and it found evidence that increased energy drink consumption was associated with attention deficit/hyperactivity disorder inattention and hyperactivity at 16-month follow-up, but did not find evidence for associations with depression, panic and anxiety [94].

## Summary of evidence

Since the most recent relevant review was published on this topic in 2009 [16], there has been a substantial increase in research examining the health consequences of SSB consumption among children and adolescents. For example, 227 studies indexed in PubMed were published on SSBs in 2017 compared to 16 studies published in 2007.^1^ Many more studies are now conducted exclusively on children and adolescents, while previous evidence was based on results found among adults. While the majority of this research is still cross-sectional (limiting the ability to make inferences about causality), the past decade has seen a growing number of longitudinal studies being implemented, as well as an increasing amount of intervention trials.

The majority of this research on SSBs over the past decade has centered on the relationship with weight gain. The findings of this review confirm that there is clear and consistent evidence that the consumption of SSBs heightens obesity risk among children and adolescents. Although a formal quality assessment or strength of evidence evaluation was not conducted, the vast majority of cross-sectional, longitudinal and intervention studies find strong evidence for a positive relationship in all or part of their study population. The exact mechanism through which SSBs impact childhood obesity is not entirely understood. Generally, the research points to the low satiety of SSBs and incomplete compensation [98, 99]. In other words, drinking calories in liquid form does not decrease hunger in the same way as solid food. Additionally, people do not sufficiently reduce their total energy intake to make up for the excess calories obtained from SSBs. There is also a lively debate about whether the effect of calories from SSBs on body weight is worse than some other foods or nutrients [100, 101].

The association between SSB consumption and weight gain is paramount, given that childhood obesity affects roughly one in six (13 million) children in the U.S., disproportionately impacting children who are low-income and racial and ethnic minorities [102]. From 1976 to 2016, the prevalence of childhood obesity in the U.S. more than doubled in children ages 2 to 5 (from 5% to 13.9%), nearly tripled in children aged 6 to 11 (from 6.5% to 18.4%) and quadrupled in adolescents’ ages 12 to 19 (from 5% to 20.6%) [103–105]. While there is some indication that childhood obesity rates may leveling in the U.S. [104], the overall prevalence of obesity among children in 2016–2016 was estimated at 18.5% [105], meaning it is still considerably higher than the Healthy People 2020 goal of 14.5% [4]. Given that children who are overweight and obese youth are likely to remain so as adults [106], obesity and its adverse health consequences create a serious threat to children’s current and future health [107]. Hence, reducing SSB consumption is an important intervention point to reduce the burden of childhood obesity in the U.S.

This review also finds strong and consistent evidence that consumption of SSBs is associated with dental caries among children and adolescents. The mechanism for the association between SSB consumption and dental caries is well understood: dental caries are caused by acids produced by bacteria metabolizing sugar in the mouth. Increased sugar from SSBs intensifies the acid production and causes further decay of teeth [108]. The majority of studies examining this relationship are cross-sectional, but a modest number of longitudinal studies as well as one intervention study also support the association.

While evidence has shown a positive relationship between SSB consumption and type 2 diabetes among adults [5, 12, 109], the available literature among child and adolescents is limited. The majority of studies among children and adolescents do not directly examine the link between SSB consumption and type 2 diabetes and instead measure insulin resistance, a biomarker of increased cardio-metabolic risk and type 2 diabetes. It is hypothesized that the high content of sucrose and high-fructose corn syrup present in SSBs may increase dietary glycemic load leading to insulin resistance and inflammation [7]. While not as strong and consistent as the relationships between SSB consumption and weight gain or dental caries, most studies in this review generally support an association between SSB consumption and insulin resistance among children and adolescents. However, this is limited by a small number of studies and the predominance of a cross-sectional study design.

The findings of this review also point to an association between caffeinated SSBs and a wide range of health issues including poor quality or reduced sleep, headaches, risk-seeking behavior and depressive symptoms. The presence of caffeine in energy drinks and other caffeinated SSBs (e.g., cola), in conjunction with the large volumes consumed, can lead to neurological and psychological effects associated with high caffeine consumption. The majority of studies examining the caffeine-related effects of SSBs focus on energy drinks, with very few analyzing the effects of other caffeinated SSBs such as colas. One reason for this may be the considerably higher level of caffeine content in energy drinks: a 250 mL energy drink has an average of 80 mg of caffeine (range: 27-87 mg), compared to 40 g of caffeine (range: 30-60 mg) in a 330 mL cola drink [110]. Additionally, studies examining caffeine-related effects have almost exclusively been cross-sectional, limiting the strength of inferences that can be made and bringing forth issues of reverse causation.

While there is a large and growing body of research examining the impact of SSBs on children’s health, important gaps remain. First, researchers should utilize more rigorous study designs (intervention trials and longitudinal studies) and move away from a reliance on cross-sectional studies. This will strengthen the evidence base and allow firmer conclusions to be made regarding the causal relationships between SSB consumption and negative health consequences. Second, more consistency is needed in the definition of SSBs (e.g., specifying which beverages are included and what is a typical serving size) and measurement strategy (e.g., FFQ vs. 24-h recall). Similarly, more uniformity is needed in assessing outcomes, particularly in the risk of overweight/obesity where studies vary considerably in the outcomes measured (e.g., BMI, BMI z-score, BMI percentile, overweight/obese status). Third, researchers should more rigorously examine differences in health risks by subpopulations (e.g., race/ethnicity, socioeconomic status, age and gender) to determine if the intake of SSBs in particularly harmful in certain population subsets. While it is established that low-income and racial and ethnic minorities consume more SSBs, it is unclear the extent to which health consequences are magnified among these groups. This is important particularly for targeting interventions and policy approaches to reduce children’s SSB consumption. Better insights in these areas have the potential to inform real-world policies and recommendations that may greatly benefit children’s health. Finally, additional research is needed about caffeinated SSBs and their impact on children’s health. Energy and sport drink consumption is rising rapidly in the U.S. [13] and so studies examining the negative health effects of caffeinated SSBs are needed to inform future efforts to reduce consumption.

This review has several limitations. First, it only focuses on four main health effects associated with SSB consumption and does not address other potential consequences which have been documented among consumers of SSBs (e.g., hyperlipidemia, non-alcoholic fatty liver disease). Second, our conclusions for a particular health consequence did not include a quality assessment and was limited to an informal evaluation of consistency and lack of conflicting studies. Third, article screening was not done in duplicate, although all included articles were confirmed by a second reviewer.

## Conclusion

This review provides clear and consistent evidence that consumption of SSBs increases obesity risk and dental caries among children and adolescents, with emerging evidence supporting an association with insulin resistance and caffeine-related effects. In general, the strength of evidence for all four health consequences could be improved through the implementation of more longitudinal and intervention studies. Additionally, more consistency is needed from studies in the measurement of exposures (e.g., standardized measurement and definition of SSBs) and outcomes (e.g., assessment of weight-related outcomes) to create a stronger evidence base. Future research should compare low-income and racial/ethnic minority subgroups in order to determine if differences in health risks associated with SSBs exist. Although SSB consumption has declined in the last 15 years, consumption still remains high (61% of children consume at least one SSB per day). The vast majority of the available literature suggests that reducing SSB consumption would improve children’s health.

## Additional file

Additional file 1: Appendix. Search Strategies (Contains the full list of search terms and PRISMA diagrams). (DOCX 128 kb)

## Abbreviations

BMI: Body mass index
NHANES: National Health and Nutritional Examination Survey
OECD: Organisation for Economic Co-operation and Development
SSB: Sugar-sweetened beverage

## Footnotes

1 Author calculations, based on PubMed results by year.
