# Supplementary material for: The negative impact of sugar-sweetened beverages on children’s health: an update of the literature
Source: BMC Obes. 2018 Feb 20;5:6. doi: 10.1186/s40608-017-0178-9 (PMC5819237; doi:10.1186/s40608-017-0178-9)
Supplement: Supplementary file 1 — Search Strategies (Contains the full list of search terms and PRISMA diagrams). (DOCX 128 kb) [file 40608_2017_178_MOESM1_ESM.docx]

**APPENDIX**

## Search Strategies

PubMed, CAB Abstracts and PAIS International were all searched. The start date restriction was Jan 1, 2007 for the searches on obesity, insulin resistance, dental caries, caffeine-related effects and substitution. There was no start date restriction for taste preferences. All searches were restricted to articles published in English.

**Obesity: searched on January 31, 2017**

PubMed

**(**("Beverages"[Mesh] OR beverage[tiab] OR beverages[tiab] OR drink[tiab] OR drinks[tiab] OR soda[tiab] OR sodas[tiab] OR juice[tiab] OR juices[tiab] OR pop[tiab] OR tea[tiab] OR teas[tiab] OR coffee*[tiab] OR milk*[tiab]) AND (("Nutritive Sweeteners"[Mesh] OR sucrose[tiab] OR fructose[tiab] OR sugar[tiab] OR sugary[tiab] OR sugared[tiab] OR sweet[tiab] OR sweetened[tiab] OR sweetener*[tiab] OR nondiet[tiab] OR non diet[tiab] OR high calorie[tiab]) OR (fruit juice*[tiab] OR fruit drink*[tiab] OR regular soda*[tiab] OR caloric beverage*[tiab] OR fruit punch*[tiab] OR sport drink*[tiab] OR energy drink*[tiab]))) AND (("Child"[Mesh] OR "Adolescent"[Mesh] OR preschooler*[tiab] OR pre schooler*[tiab] OR pre school[tiab] OR preschool[tiab] OR toddler*[tiab] OR child[tiab] OR children[tiab] OR children’s[tiab] OR childhood[tiab] OR adolescent[tiab] OR adolescents[tiab] OR adolescence[tiab] OR teen[tiab] OR teens[tiab] OR teenager[tiab] OR teenagers[tiab] OR teenage[tiab] OR prekindergar*[tiab] OR nursery school*[tiab] OR head start[tiab] OR kindergar*[tiab] OR grade school*[tiab] OR elementary school*[tiab] OR elementary student*[tiab] OR middle school*[tiab] OR high school*[tiab] OR highschool*[tiab] OR public school*[tiab] OR boy[tiab] OR boys[tiab] OR girl[tiab] OR girls[tiab] OR pediatric*[tiab] OR paediatric*[tiab])) AND ((“Overweight” [Mesh] OR “Body Weight” [Mesh:NoExp] OR “Body Weight Changes” [Mesh] OR “Body Composition” [Mesh] OR “Body Mass Index” [Mesh] OR obese[tiab] OR obesity[tiab] OR overweight[tiab] OR weight[tiab] OR weighed[tiab] OR body mass[tiab] OR bmi[tiab] OR body composition[tiab] OR adipose[tiab] OR adiposity[tiab]))

CAB Abstracts and PAIS International

((beverage OR beverages OR drink OR drinks OR soda OR sodas OR juice OR juices OR pop OR tea OR teas OR coffee* OR milk*) AND (("Nutritive Sweeteners" OR sucrose OR fructose OR sugar OR sugary OR sugared OR sweet OR sweetened OR sweetener* OR nondiet OR “non diet” OR “high calorie”) OR (“fruit juice*” OR “fruit drink*” OR “regular soda*” OR “caloric beverage*” OR “fruit punch*” OR “sport drink*” OR “energy drink*”))) AND (preschooler* OR “pre schooler*” OR “pre school” OR preschool OR toddler* OR child OR children OR children’s OR childhood OR adolescent OR adolescents OR adolescence OR teen OR teens OR teenager OR teenagers OR teenage OR prekindergar* OR nursery school* OR “head start” OR kindergar* OR “grade school*” OR “elementary school*” OR “elementary student*” OR “middle school*” OR “high school*” OR highschool* OR “public school*” OR boy OR boys OR girl OR girls OR pediatric* OR paediatric*) AND (obese OR obesity OR overweight OR weight OR weighed OR “body mass” OR bmi OR “body composition” OR adipose OR adiposity)

**Insulin Resistance: searched on February 16, 2017**

PubMed

((((("Beverages"[Mesh] OR beverage[tiab] OR beverages[tiab] OR drink[tiab] OR drinks[tiab] OR soda[tiab] OR sodas[tiab] OR juice[tiab] OR juices[tiab] OR pop[tiab] OR tea[tiab] OR teas[tiab] OR coffee*[tiab] OR milk*[tiab]) AND ("Nutritive Sweeteners"[Mesh] OR sucrose[tiab] OR fructose[tiab] OR sugar[tiab] OR sugary[tiab] OR sugared[tiab] OR sweet[tiab] OR sweetened[tiab] OR sweetener*[tiab] OR nondiet[tiab] OR non diet[tiab] OR high calorie[tiab]) OR (fruit juice*[tiab] OR fruit drink*[tiab] OR regular soda*[tiab] OR caloric beverage*[tiab] OR fruit punch*[tiab] OR sport drink*[tiab] OR energy drink*[tiab])))) AND (("Child"[Mesh] OR "Adolescent"[Mesh] OR preschooler*[tiab] OR pre schooler*[tiab] OR pre school[tiab] OR preschool[tiab] OR toddler*[tiab] OR child[tiab] OR children[tiab] OR children’s[tiab] OR childhood[tiab] OR adolescent[tiab] OR adolescents[tiab] OR adolescence[tiab] OR teen[tiab] OR teens[tiab] OR teenager[tiab] OR teenagers[tiab] OR teenage[tiab] OR prekindergar*[tiab] OR nursery school*[tiab] OR head start[tiab] OR kindergar*[tiab] OR grade school*[tiab] OR elementary school*[tiab] OR elementary student*[tiab] OR middle school*[tiab] OR high school*[tiab] OR highschool*[tiab] OR public school*[tiab] OR boy[tiab] OR boys[tiab] OR girl[tiab] OR girls[tiab] OR pediatric*[tiab] OR paediatric*[tiab]))) AND (("Diabetes Mellitus"[Mesh] OR "Insulin Resistance"[MeSH] OR diabetes[tiab] OR diabetic*[tiab] OR insulin resistance[tiab] OR type 2 DM [tiab] OR type II DM [tiab]))

CAB Abstracts and PAIS International

((beverage OR beverages OR drink OR drinks OR soda OR sodas OR juice OR juices OR pop OR tea OR teas OR coffee* OR milk*) AND (("Nutritive Sweeteners" OR sucrose OR fructose OR sugar OR sugary OR sugared OR sweet OR sweetened OR sweetener* OR nondiet OR “non diet” OR “high calorie”) OR (“fruit juice*” OR “fruit drink*” OR “regular soda*” OR “caloric beverage*” OR “fruit punch*” OR “sport drink*” OR “energy drink*”))) AND (preschooler* OR “pre schooler*” OR “pre school” OR preschool OR toddler* OR child OR children OR children’s OR childhood OR adolescent OR adolescents OR adolescence OR teen OR teens OR teenager OR teenagers OR teenage OR prekindergar* OR nursery school* OR “head start” OR kindergar* OR “grade school*” OR “elementary school*” OR “elementary student*” OR “middle school*” OR “high school*” OR highschool* OR “public school*” OR boy OR boys OR girl OR girls OR pediatric* OR paediatric*) AND (diabetes OR diabetic* OR “insulin resistance” OR “type 2 DM” OR “type II DM”)

**Dental Caries: searched on March 1, 2017**

PubMed

(("Beverages"[Mesh] OR beverage[tiab] OR beverages[tiab] OR drink[tiab] OR drinks[tiab] OR soda[tiab] OR sodas[tiab] OR juice[tiab] OR juices[tiab] OR pop[tiab] OR tea[tiab] OR teas[tiab] OR coffee*[tiab] OR milk*[tiab]) AND (("Nutritive Sweeteners"[Mesh] OR sucrose[tiab] OR fructose[tiab] OR sugar[tiab] OR sugary[tiab] OR sugared[tiab] OR sweet[tiab] OR sweetened[tiab] OR sweetener*[tiab] OR nondiet[tiab] OR non diet[tiab] OR high calorie[tiab]) OR (fruit juice*[tiab] OR fruit drink*[tiab] OR regular soda*[tiab] OR caloric beverage*[tiab] OR fruit punch*[tiab] OR sport drink*[tiab] OR energy drink*[tiab]))) AND (("Child"[Mesh] OR "Adolescent"[Mesh] OR preschooler*[tiab] OR pre schooler*[tiab] OR pre school[tiab] OR preschool[tiab] OR toddler*[tiab] OR child[tiab] OR children[tiab] OR children’s[tiab] OR childhood[tiab] OR adolescent[tiab] OR adolescents[tiab] OR adolescence[tiab] OR teen[tiab] OR teens[tiab] OR teenager[tiab] OR teenagers[tiab] OR teenage[tiab] OR prekindergar*[tiab] OR nursery school*[tiab] OR head start[tiab] OR kindergar*[tiab] OR grade school*[tiab] OR elementary school*[tiab] OR elementary student*[tiab] OR middle school*[tiab] OR high school*[tiab] OR highschool*[tiab] OR public school*[tiab] OR boy[tiab] OR boys[tiab] OR girl[tiab] OR girls[tiab] OR pediatric*[tiab] OR paediatric*[tiab])) AND ("Dental Caries"[Mesh:NoExp] OR caries[tiab] OR tooth decay[tiab] OR dental decay[tiab])

Cab Abstracts and PAIS International

((beverage OR beverages OR drink OR drinks OR soda OR sodas OR juice OR juices OR pop OR tea OR teas OR coffee* OR milk*) AND (("Nutritive Sweeteners" OR sucrose OR fructose OR sugar OR sugary OR sugared OR sweet OR sweetened OR sweetener* OR nondiet OR “non diet” OR “high calorie”) OR (“fruit juice*” OR “fruit drink*” OR “regular soda*” OR “caloric beverage*” OR “fruit punch*” OR “sport drink*” OR “energy drink*”))) AND (preschooler* OR “pre schooler*” OR “pre school” OR preschool OR toddler* OR child OR children OR children’s OR childhood OR adolescent OR adolescents OR adolescence OR teen OR teens OR teenager OR teenagers OR teenage OR prekindergar* OR nursery school* OR “head start” OR kindergar* OR “grade school*” OR “elementary school*” OR “elementary student*” OR “middle school*” OR “high school*” OR highschool* OR “public school*” OR boy OR boys OR girl OR girls OR pediatric* OR paediatric*) AND (caries OR tooth decay OR dental decay)

**Caffeine-related: searched on March 19, 2017**

PubMed

((((("Beverages"[Mesh] OR beverage[tiab] OR beverages[tiab] OR drink[tiab] OR drinks[tiab] OR soda[tiab] OR sodas[tiab] OR juice[tiab] OR juices[tiab] OR pop[tiab] OR tea[tiab] OR teas[tiab] OR coffee*[tiab] OR milk*[tiab]) AND ("Nutritive Sweeteners"[Mesh] OR sucrose[tiab] OR fructose[tiab] OR sugar[tiab] OR sugary[tiab] OR sugared[tiab] OR sweet[tiab] OR sweetened[tiab] OR sweetener*[tiab] OR nondiet[tiab] OR non diet[tiab] OR high calorie[tiab]) OR (fruit juice*[tiab] OR fruit drink*[tiab] OR regular soda*[tiab] OR caloric beverage*[tiab] OR fruit punch*[tiab] OR sport drink*[tiab] OR energy drink*[tiab])))) AND (("Child"[Mesh] OR "Adolescent"[Mesh] OR preschooler*[tiab] OR pre schooler*[tiab] OR pre school[tiab] OR preschool[tiab] OR toddler*[tiab] OR child[tiab] OR children[tiab] OR children’s[tiab] OR childhood[tiab] OR adolescent[tiab] OR adolescents[tiab] OR adolescence[tiab] OR teen[tiab] OR teens[tiab] OR teenager[tiab] OR teenagers[tiab] OR teenage[tiab] OR prekindergar*[tiab] OR nursery school*[tiab] OR head start[tiab] OR kindergar*[tiab] OR grade school*[tiab] OR elementary school*[tiab] OR elementary student*[tiab] OR middle school*[tiab] OR high school*[tiab] OR highschool*[tiab] OR public school*[tiab] OR boy[tiab] OR boys[tiab] OR girl[tiab] OR girls[tiab] OR pediatric*[tiab] OR paediatric*[tiab]))) AND ("Caffeine"[Mesh] OR caffeine[tiab] OR caffeinated[tiab])

CAB Abstracts and PAIS International

((beverage OR beverages OR drink OR drinks OR soda OR sodas OR juice OR juices OR pop OR tea OR teas OR coffee* OR milk*) AND (("Nutritive Sweeteners" OR sucrose OR fructose OR sugar OR sugary OR sugared OR sweet OR sweetened OR sweetener* OR nondiet OR “non diet” OR “high calorie”) OR (“fruit juice*” OR “fruit drink*” OR “regular soda*” OR “caloric beverage*” OR “fruit punch*” OR “sport drink*” OR “energy drink*”))) AND (preschooler* OR “pre schooler*” OR “pre school” OR preschool OR toddler* OR child OR children OR children’s OR childhood OR adolescent OR adolescents OR adolescence OR teen OR teens OR teenager OR teenagers OR teenage OR prekindergar* OR nursery school* OR “head start” OR kindergar* OR “grade school*” OR “elementary school*” OR “elementary student*” OR “middle school*” OR “high school*” OR highschool* OR “public school*” OR boy OR boys OR girl OR girls OR pediatric* OR paediatric*) AND (caffeine OR caffeinated)

**Substitution Effects: searched on March 11, 2017**

PubMed

(("Beverages"[Mesh] OR beverage[tiab] OR beverages[tiab] OR drink[tiab] OR drinks[tiab] OR soda[tiab] OR sodas[tiab] OR juice[tiab] OR juices[tiab] OR pop[tiab] OR tea[tiab] OR teas[tiab] OR coffee*[tiab] OR milk*[tiab]) AND (("Nutritive Sweeteners"[Mesh] OR sucrose[tiab] OR fructose[tiab] OR sugar[tiab] OR sugary[tiab] OR sugared[tiab] OR sweet[tiab] OR sweetened[tiab] OR sweetener*[tiab] OR nondiet[tiab] OR non diet[tiab] OR high calorie[tiab]) OR (fruit juice*[tiab] OR fruit drink*[tiab] OR regular soda*[tiab] OR caloric beverage*[tiab] OR fruit punch*[tiab] OR sport drink*[tiab] OR energy drink*[tiab]))) AND (("Child"[Mesh] OR "Adolescent"[Mesh] OR preschooler*[tiab] OR pre schooler*[tiab] OR pre school[tiab] OR preschool[tiab] OR toddler*[tiab] OR child[tiab] OR children[tiab] OR children’s[tiab] OR childhood[tiab] OR adolescent[tiab] OR adolescents[tiab] OR adolescence[tiab] OR teen[tiab] OR teens[tiab] OR teenager[tiab] OR teenagers[tiab] OR teenage[tiab] OR prekindergar*[tiab] OR nursery school*[tiab] OR head start[tiab] OR kindergar*[tiab] OR grade school*[tiab] OR elementary school*[tiab] OR elementary student*[tiab] OR middle school*[tiab] OR high school*[tiab] OR highschool*[tiab] OR public school*[tiab] OR boy[tiab] OR boys[tiab] OR girl[tiab] OR girls[tiab] OR pediatric*[tiab] OR paediatric*[tiab])) AND ("Non-Nutritive Sweeteners"[Mesh] OR artificially sweetened[tiab] OR non caloric[tiab] OR sugar free[tiab] OR diet soda*[tiab] OR diet drink*[tiab] OR diet soft drink*[tiab] OR diet beverage*[tiab] OR water[tiab] OR waters[tiab] OR milk[tiab] OR milks[tiab]) AND (substitute[tiab] OR substitution*[tiab] OR substituting[tiab] OR replace[tiab] OR replaced[tiab] OR replacement[tiab] OR replacing[tiab] OR switch[tiab] OR switched[tiab] OR switching[tiab])

CAB Abstracts and PAIS International

((beverage OR beverages OR drink OR drinks OR soda OR sodas OR juice OR juices OR pop OR tea OR teas OR coffee* OR milk*) AND (("Nutritive Sweeteners" OR sucrose OR fructose OR sugar OR sugary OR sugared OR sweet OR sweetened OR sweetener* OR nondiet OR “non diet” OR “high calorie”) OR (“fruit juice*” OR “fruit drink*” OR “regular soda*” OR “caloric beverage*” OR “fruit punch*” OR “sport drink*” OR “energy drink*”))) AND (preschooler* OR “pre schooler*” OR “pre school” OR preschool OR toddler* OR child OR children OR children’s OR childhood OR adolescent OR adolescents OR adolescence OR teen OR teens OR teenager OR teenagers OR teenage OR prekindergar* OR nursery school* OR “head start” OR kindergar* OR “grade school*” OR “elementary school*” OR “elementary student*” OR “middle school*” OR “high school*” OR highschool* OR “public school*” OR boy OR boys OR girl OR girls OR pediatric* OR paediatric*) AND (artificially sweetened OR non caloric OR sugar free OR diet soda* OR diet drink* OR diet soft drink* OR diet beverage* OR water OR waters OR milk OR milks) AND (substitute OR substitution* OR substituting OR replace OR replaced OR replacement OR replacing OR switch OR switched OR switching)

##
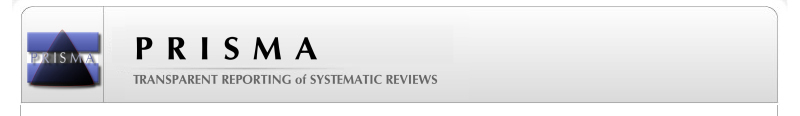
PRISMA Flow Diagram: Obesity

Records identified through database searching
(n = 3488 )

**PubMed:** 1551

**Cab Abstracts:** 1900

**PAIS International:** 37

Titles and Abstracts screened (n = 2854 )
(n = 2816 )

Records after duplicates removed
(n = 2854 )

Additional records identified through other sources
(n = 1 )

Titles and abstracts excluded
(n = 2675 )

Included Studies
(n = 41 )

Full-text screened
(n =179 )

Full-text excluded
(n = 138 )

Not original data/review = 28

Not examining SSB consumption = 13

Not examining obesity = 20

Not examining effect of SSBs on obesity= 2

Non-OECD country = 22

Age = 2

SSB consumption is not primary risk factor = 51

##
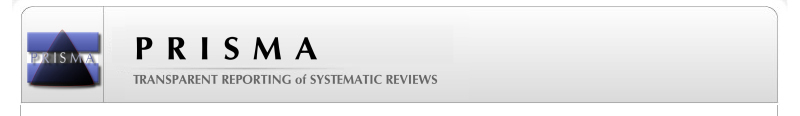
PRISMA Flow Diagram: Insulin Resistance

Records identified through database searching
(n = 375 )

**PubMed:** 207

**Cab Abstracts:** 164

**PAIS International:** 4

Titles and Abstracts screened (n = 308 )
(n = 2816 )

Records after duplicates removed
(n = 308 )

Titles and abstracts excluded
(n = 280 )

Included Studies
(n = 6 )

Full-text screened
(n =28 )

Full-text excluded
(n = 22 )

Not original data/review = 13

Not examining SSB consumption = 3

Not examining insulin resistance = 2

Non-OECD country = 2

Age = 2

##
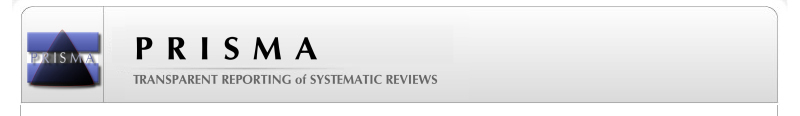
PRISMA Flow Diagram: Dental Caries

Records identified through database searching
(n = 334 )

**PubMed:** 214

**Cab Abstracts:** 117

**PAIS International:** 3

Titles and Abstracts screened (n = 275 )
(n = 2816 )

Records after duplicates removed
(n = 275 )

Titles and abstract excluded
(n = 218 )

Included Studies
(n = 23 )

Full-text screened
(n =57 )

Full-text excluded
(n = 34 )

Not original data/review = 9

Not examining SSB consumption = 17

Not examining dental caries = 2

Non-OECD country = 3

Not examining effect of SSBs on dental caries = 2

Language = 1

##
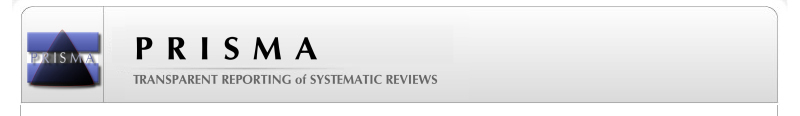
PRISMA Flow Diagram: Caffeine-related

Records identified through database searching
(n = 279 )

**PubMed:** 184

**Cab Abstracts:** 93

**PAIS International:** 2

Titles and Abstracts screened (n = 212 )
(n = 2816 )

Records after duplicates removed
(n = 212 )

Titles and abstract excluded
(n = 188 )

Included Studies
(n = 7 )

Full-text screened
(n =24 )

Full-text excluded
(n = 17 )

Not original data/review = 11

Not examining SSB consumption = 1

Not examining caffeine-related= 1

Not examining effect of SSBs on caffeine-related issues = 2

Age = 2

##
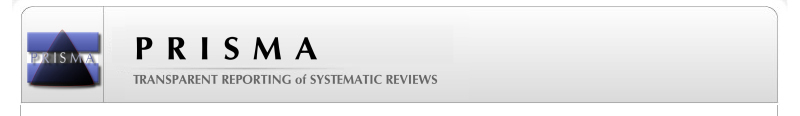
PRISMA Flow Diagram: Substitution

Records identified through database searching
(n = 166 )

**PubMed:** 70

**Cab Abstracts:** 94

**PAIS International:** 2

Titles and Abstracts screened (n = 134 )
(n = 2816 )

Records after duplicates removed
(n = 134 )

Titles and abstract excluded
(n = 114 )

Included Studies
(n = 10 )

Full-text screened
(n =20 )

Full-text excluded
(n = 10 )

Not examining substitution = 1

Not examining relevant outcomes = 6

Age = 3
